# Vertical Binocular Disparity is Encoded Implicitly within a Model Neuronal Population Tuned to Horizontal Disparity and Orientation

**DOI:** 10.1371/journal.pcbi.1000754

**Published:** 2010-04-22

**Authors:** Jenny C. A. Read

**Affiliations:** Institute of Neuroscience, Newcastle University, Newcastle upon Tyne, United Kingdom; New York University, United States of America

## Abstract

Primary visual cortex is often viewed as a “cyclopean retina”, performing the initial encoding of binocular disparities between left and right images. Because the eyes are set apart horizontally in the head, binocular disparities are predominantly horizontal. Yet, especially in the visual periphery, a range of non-zero vertical disparities do occur and can influence perception. It has therefore been assumed that primary visual cortex must contain neurons tuned to a range of vertical disparities. Here, I show that this is not necessarily the case. Many disparity-selective neurons are most sensitive to changes in disparity orthogonal to their preferred orientation. That is, the disparity tuning surfaces, mapping their response to different two-dimensional (2D) disparities, are elongated along the cell's preferred orientation. Because of this, even if a neuron's optimal 2D disparity has zero vertical component, the neuron will still respond best to a non-zero vertical disparity when probed with a sub-optimal horizontal disparity. This property can be used to decode 2D disparity, even allowing for realistic levels of neuronal noise. Even if all V1 neurons at a particular retinotopic location are tuned to the expected vertical disparity there (for example, zero at the fovea), the brain could still decode the magnitude and sign of departures from that expected value. This provides an intriguing counter-example to the common wisdom that, in order for a neuronal population to encode a quantity, its members must be tuned to a range of values of that quantity. It demonstrates that populations of disparity-selective neurons encode much richer information than previously appreciated. It suggests a possible strategy for the brain to extract rarely-occurring stimulus values, while concentrating neuronal resources on the most commonly-occurring situations.

## Author Summary

Because our eyes are set apart horizontally in our head, the images they see are mainly offset horizontally. However, small vertical disparities also occur, and can have a measurable effect on perception, showing that they must be detected by the visual system. The trouble is that encoding a two-dimensional quantity is much more expensive for neuronal systems than encoding a one-dimensional quantity. This paper shows that, for two-dimensional disparity, the brain could potentially take advantage of a major simplification. This strategy would avoid the need to build neurons tuned to a range of vertical disparities at each retinotopic location. For example, at the centre of the visual field, vertical disparities are almost always zero. The brain could make sure all its neurons at this location respond best to zero vertical disparity, ensuring best performance for the most common disparities. But the brain would still know what the vertical disparity actually was, which would be useful on rare occasions where it was not zero, e.g., when the eyes are misaligned. This is an interesting example because usually, neuronal populations which are all tuned to the same value of a quantity cannot encode that quantity (e.g., a retina with only one type of cone cell cannot encode color).

## Introduction

It is commonly accepted that in order for a neuronal population to encode the value of a quantity x, it must contain cells tuned to a range of values of x. Thus for example the retina can encode information about the wavelength of light because it contains three different types of cones with different tuning to wavelength, and the primary visual cortex can encode feature orientation because it contains neurons tuned to a range of orientations. This is unproblematic because natural images contain a wide range of light wavelengths and object orientations. However, the same argument applied to stereo vision produces some more challenging conclusions.

The expected vertical disparity in natural viewing depends on position in the retina, with opposite signs in opposite quadrants of the visual field. The range in vertical disparities encountered at a given position depends on a number of assumptions about eye movement and scene statistics, but all attempts to estimate it agree that it is extremely narrowly distributed compared to horizontal disparity [1], [2], [3]. Thus, if disparity sensors in the brain were to reflect disparity in the natural world, we would expect the distribution of two-dimensional disparity tuning at a given retinotopic location to be highly elongated, virtually one-dimensional, with a wide range of horizontal disparity and a narrow range of vertical disparity, centered on the value expected for that retinotopic location. Yet, vertical disparities which hardly ever occur in normal visual experience can still have demonstrable effects on perception in the lab [4], [5], and there is evidence that stereo matching occurs in all 2D directions, vertical as well as horizontal [6]. Thus, the brain clearly can extract unusual vertical disparities, on relatively local scales [7], [8], [9]. This has led to the conclusion that the brain must contain neurons tuned to a range of vertical disparities, including highly unusual ones, on the assumption that otherwise, these disparities could not be perceived [10], [11], [12].

Motivated by this, a number of physiological studies have examined two-dimensional disparity tuning in cortical neurons in monkey primary visual cortex (V1). Near the fovea, most disparity-tuned neurons are tuned to vertical disparities which are not significantly different from zero, given the confidence interval on the measurement [13]. In the visual periphery, neurons tuned to non-zero vertical disparities have been reported [10], [11], [12]. Unfortunately, these studies only reported disparity in head-centric coordinates, which can differ substantially from retino-centric disparity [14]. For example, it is perfectly possible for a neuron tuned to a substantial head-centric vertical disparity, say 0.3°, to be tuned to a vertical disparity of 0° on the retina [3]. Thus, the published data do not enable us to draw any conclusions about 2D disparity tuning on the retina. Furthermore, these studies did not report the retinal location of individual neurons, making it impossible to assess whether a range of vertical disparity tuning is found at a single retinotopic location.

**Figure 1:**
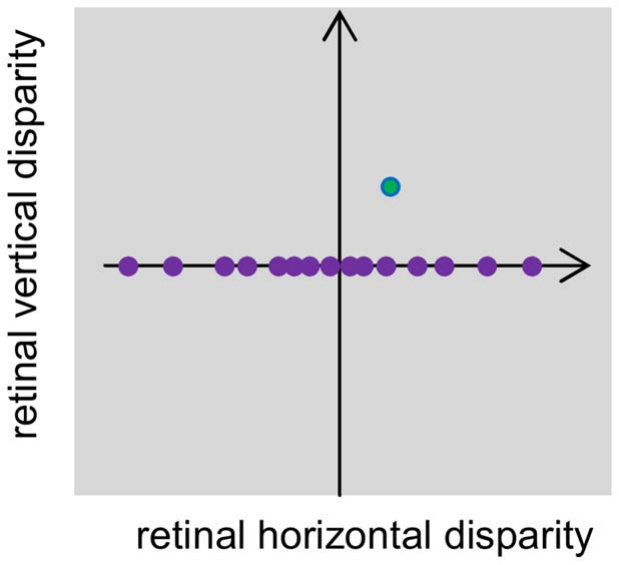
A neuronal population which explicitly encodes horizontal, but not vertical, disparity. The shaded region represents the space of two-dimensional disparity on the retina [14]. The purple disks represent the preferred 2D disparity of an idealized population of disparity sensors. Although these sensors form a one-dimensional population, all tuned to zero vertical disparity, they can nevertheless encode two-dimensional stimulus disparity, e.g. the stimulus disparity represented by the green dot, which has both a horizontal and a vertical component. (Cf figure 1 of Serrano-Pedraza & Read [16].)

Given this lack of data from physiology, theoretical considerations become important. A clear understanding of how, in principle, neurons could represent two-dimensional disparity is essential for guiding future physiology experiments. We recently argued [15] that a population of model binocular neurons like that shown in Figure 1, tuned to a range of horizontal disparities and orientations but all tuned to zero vertical disparity on the retina, nevertheless encodes information about the vertical disparity of the stimulus. This original model only extracted the magnitude, not the sign, of the local vertical disparity, and we later demonstrated that this was inconsistent with human psychophysics [16]. However, this model did not make optimal use of the information available in the population. In the present paper, I show that this population of disparity sensors does contain information about both the magnitude and the sign of the vertical disparity at that point in the retina, even if all neurons in the population are tuned to the same vertical disparity. With an appropriate decoding technique, information about the two-dimensional disparity can be deduced from activity in this one-dimensional population. This result is of interest in its own right as a theoretical demonstration that it is possible to extract the value of a quantity from a neuronal population, all of whose members respond optimally to the same value of that quantity. From the point of view of understanding stereo vision, it means that two-dimensional disparity may be represented far more efficiently than previously appreciated.

## Methods

### Overview

**Figure 2:**
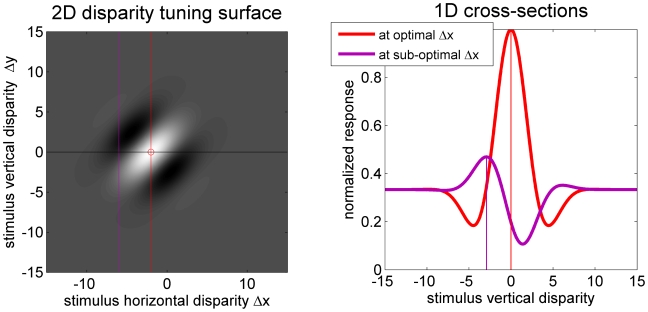
Cells with obliquely oriented 2D disparity tuning surfaces are tuned to non-zero vertical disparities at non-optimal horizontal disparities. A: 2D disparity tuning surface. The preferred 2D disparity is marked with a red circle: it has no vertical component. B: 1D disparity tuning curves showing neuron's response to vertical disparity, at the horizontal disparities marked with the red and purple lines in A. At the non-optimal horizontal disparity (purple curve), the neuron responds best to non-zero vertical disparities.

The essential insight guiding this paper is relatively trivial. According to the stereo energy model of disparity-selective neurons [17], [18], cells with obliquely-oriented receptive fields will also have obliquely-oriented disparity tuning surfaces, like the one illustrated in Figure 2A. This cell's optimal disparity is marked with a red circle. It has zero vertical component, i.e. the cell responds best to zero vertical disparity. Figure 2B shows two cross-sections through this surface, corresponding to vertical disparity tuning curves for two different horizontal disparities, as indicated by the vertical lines in Figure 2A. At the optimal horizontal disparity (red curve), the cell responds best to zero vertical disparity. But at horizontal disparities away from the optimum (e.g. purple curve), the cell's response is reduced, but is now tuned to a non-zero vertical disparity. Thus, while the cell in Figure 2 is “tuned to zero vertical disparity” in that its optimum 2D disparity has zero vertical component, when it is probed at horizontal disparities on either side of the optimum, it responds best to vertical disparities on either side of zero. This suggests that, given cells tuned to a range of orientations and horizontal disparities, one could potentially extract the stimulus orientation, horizontal disparity *and* vertical disparity. Of course, it may not be quite that simple. In order to use the cells' tuning to vertical disparity away from the optimal horizontal disparity, one has to know what the horizontal disparity is. Extracting this may be hard in the presence of vertical disparity, since then none of the cells in the population is tuned to the correct stimulus disparity. Also, because the tuning to vertical disparity occurs only at sub-optimal horizontal disparities, the neuron's activity is weaker, so more subject to noise. Thus, this intuitive idea has to be rigorously tested by simulation. This is what is achieved in this paper.

The simulations consist of two neuronal populations: one encoding population, which takes left and right retinal images and performs the initial encoding of binocular disparity, and one decoding population, which estimates the disparity of the stimulus. The encoding population is like that in Figure 1: it consists of a set of neurons tuned to a range of horizontal disparities, orientations and spatial frequencies, but all tuned to the same vertical disparity. For simplicity, I shall set this vertical disparity to be zero, which is appropriate for the parafoveal region.

The encoding neurons are based on the stereo energy model [17], normalized so as to report the effective local binocular correlation [15], [19], [20]. The activity of this population is then decoded by a separate, higher-level population, using a template-matching approach like that of Tsai & Victor [21]. The synaptic weights from the encoding to the decoding population store the mean response of the population to stimuli with a range of different two-dimensional disparities. To estimate the two-dimensional disparity of a test image, I simply calculate the correlation between the population response to the test image, and the stored average population response for each known 2D disparity. The stimulus disparity is taken to be that giving the highest correlation, i.e. the best match to the mean response.

### Disparity encoding

#### Receptive fields

**Figure 3:**
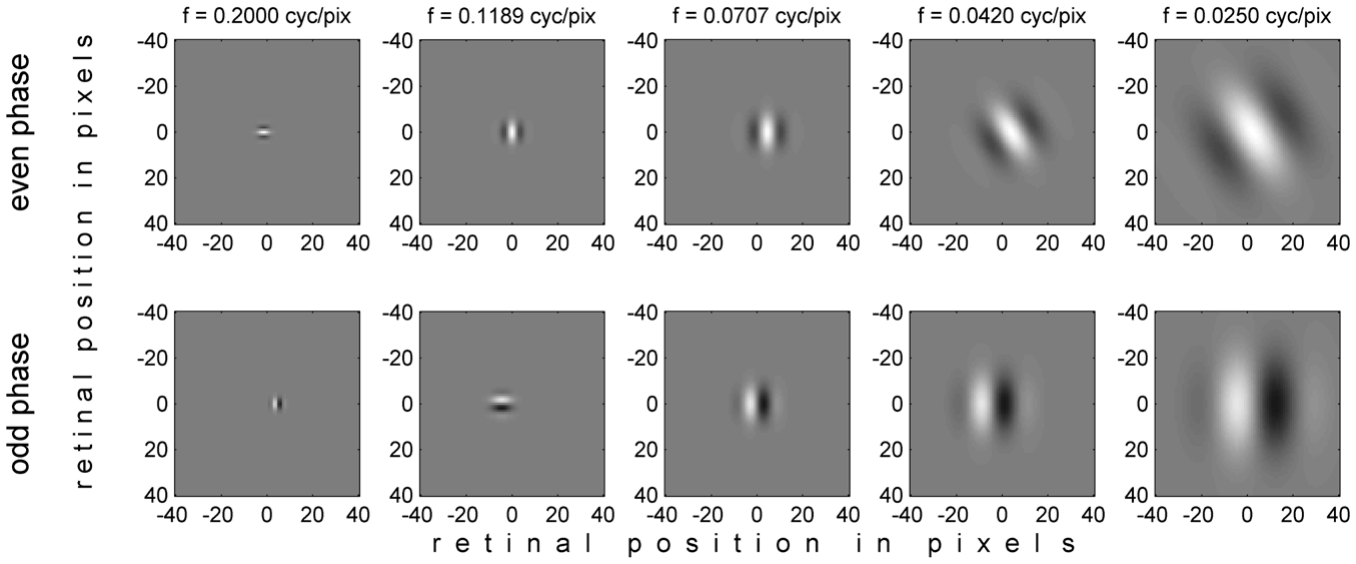
Example receptive fields in the two eyes. The columns show the 5 different spatial frequencies, f; the receptive field envelope σ was set to 0.25/f. The two rows show 2 different phases φ: top row, even phase (φ = 0), bottom row, odd phase (φ = π/2). θ and Δx are chosen randomly in each plot from the values included in the population. Matlab code to generate this figure is Protocol S1.

The monocular receptive fields were Gabor functions varying in their preferred orientation θ, spatial frequency f, receptive field size σ, receptive field phase φ, and position on the retina (Figure 3). The two receptive fields of a given binocular neurons always had the same orientation, frequency and size, but could differ in their phase and position, reflecting the properties of real neurons in primary visual cortex [22], [23], [24], [25], [26]. Thus, the model binocular simple cells in general had both position and phase disparity [22]. All model binocular simple cells were tuned to the same cyclopean position, which was the origin. That is, the mean of the receptive field centers in the left and right eyes was (0,0) for all cells.

The aim of this study is to demonstrate that vertical disparity can be implicitly encoded by a population of neurons that are all tuned to a single vertical disparity. Here, I choose this single vertical disparity tuning to be zero, reflecting the vertical disparity expected at the fovea, (0,0). At other retinotopic locations, a different value would be appropriate, reflecting the expected vertical disparity at that location [14]. The particular value chosen is not important to the demonstration, only the fact that it is the same for all neurons in the population. Including phase disparity in the model makes this slightly more complicated, since for neurons tuned to non-vertical orientations, phase disparity adds both a horizontal and a vertical component to the preferred disparity. To deal with this, each neuron is given a position disparity chosen to cancel out the component introduced by the phase disparity. Thus, even in considering a single neuron, there are several different meanings of disparity to distinguish. In this paper, Δ*x*_enc_ will indicate the preferred horizontal disparity of an encoding neuron, i.e. the horizontal disparity which elicits its maximum firing rate (the preferred vertical disparity of all encoding neurons is Δ*y*_enc_ = 0). Δφ indicates the phase disparity of an encoding neuron. Finally (Δ*x*_pos_,Δ*y*_pos_) indicates the two-dimensional position disparity, chosen to be
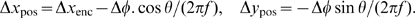
(1)For sufficiently narrow-band cells, this ensures that the neuron is tuned to the desired horizontal disparity of Δ*x*_enc_, and to zero vertical disparity.

The left and right eye receptive fields of the binocular simple cell tuned to orientation θ, frequency f, receptive field size σ, phase φ and horizontal disparity Δx are then
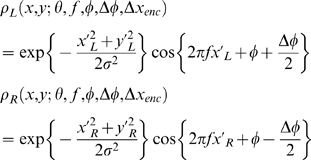
(2)where x′ and y′ are retinal coordinates offset to the centre of the receptive field, and rotated to line up with the cell's preferred orientation:
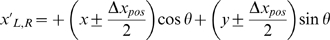


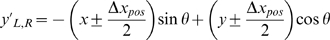
taking the + signs for *x*′_L_, *y*′_L_, and the − minus signs for *x*′_R_, *y*′_R_, and where the position disparity (Δ*x*_pos_,Δ*y*_pos_) is as specified in Equation 1.

The population included a range of values for preferred orientation θ, spatial frequency *f*, receptive field size σ, phase φ, phase disparity Δφ and horizontal disparity Δ*x*_enc_ , as follows:

Orientation θ: 6 values, −60°, −30°, 0°, 30°, 60° and 90°. 90° is horizontal, 0° is vertical.
Phase φ: 2 values, 0 or π/2 (this is all that is needed to achieve a phase-invariant complex cell)
Horizontal position disparity Δ*x*_enc_: 21 values, −10 to 10 pixels in steps of 1 pixel.
Spatial frequency: 5 values, 0.200, 0.112, 0.0707, 0.0420, 0.0250 cycles per pixel, corresponding to spatial periods λ of 5.00, 8.41, 14.14, 23.81, 40.00 pixels. Receptive field size σ was set equal to 0.35λ.
Phase disparity Δφ: 5 values, 0, ±π/4 and ±π/2.

Thus, there were 6×2×21×5×5 = 6300 binocular simple cells. These values were chosen to maximize physiological plausibility while giving reasonable simulation run-times. The different parameters have different effects on the model's performance. Self-evidently, sensitivity to a range of horizontal disparities is essential. The model's ability to extract the sign of vertical disparity depends on neurons tuned to oblique orientations (Figure 2). A range of spatial frequencies is not required for the model to extract vertical disparity in principle, but does improve the range of vertical disparity magnitudes over which the model performs well. For small vertical disparities, neurons tuned to high spatial frequencies are most sensitive to the disparity. For large vertical disparities, it is neurons tuned to low spatial frequencies which are most informative, since only these have receptive fields large enough to detect the disparity. A range of phase and phase disparity is not necessary for the model to work in principle, but helps to improve the model's accuracy [27].

#### Stereo energy model

The output from each receptive field was taken to be the inner product of each eye's image I(x,y) with the corresponding receptive field:
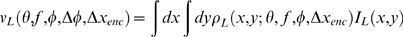
and similarly for *v*_R_. *I*(*x*,*y*) represents the contrast of the image at the point (*x*,*y*) relative to the mean luminance: positive values represent bright pixels, and negative values dark ones. In the standard energy model [17], [18], [28], [29], the response of binocular simple cells would be
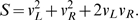
It will be convenient to split this into monocular and binocular terms:
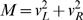


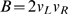
Energy-model complex cells, which are invariant to stimulus phase, are built by summing the response of binocular simple cells tuned to different phases:
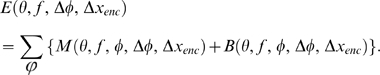
(3)As noted in the previous section, my population of simple cells includes only two values of phase, 90° apart. This produces the same results as summing over large number of simple cells with randomly-scattered phase, and is thus a widely-used short-cut in simulating complex-cell responses [28], [30], [31].

The stereo energy, E, represents something close to the cross-correlation function between the filtered, windowed images. The problem with using this to extract stimulus disparity is that it reflects not only the degree of similarity between the shifted left- and right-eye images, but also their monocular contrast energy. Thus an energy-model unit may respond strongly either because it is genuinely tuned to the stimulus disparity, or because both its monocular receptive fields happen to contain features which drive them well – whether or not those features match between the eyes. This makes it difficult to extract stimulus disparity from the stereo energy computed in Equation 3.

#### Effective binocular correlation

To overcome this, I based my template-matching on the response of normalized correlation detectors [15], [19], [20]. These are based on the stereo energy model, but are normalized so that their response ranges between +1 (when the left and right images are identical), and −1 (when the left image is an inverted version of the right). This is achieved by dividing the binocular terms of the energy-model complex cell by the monocular terms:
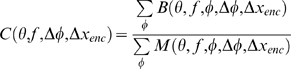
(4)Physiologically, this could be computed by combining the outputs of energy-model neurons with phase-disparities π apart. If two neurons are identical except that their phase-disparities are π apart, then if the first neuron computes E = (M+B), the second will compute (M−B). M and B are then available from the sum and difference of this pair of neurons. Thus the simulations implicitly use the full range of phase disparity, even though only phase disparity in the range [−π/2,+π/2] is explicitly simulated.

The quantity *C* computes the correlation coefficient between filtered, local regions of the left and right eye's images [27]. It can be thought of as the effective binocular correlation experienced by that cell, and takes values in the range [−1,1]. To avoid any later confusion, note that this correlation is quite distinct from the Pearson product-moment correlation coefficient used below to assess how well population activity elicited by a test stimulus matches a template.

I view the population of binocular correlation detectors, *C*(*θ*, *f*,Δφ,Δ*x*_enc_), as performing the initial encoding of disparity within my model. Recall that there are 6 different orientations, 5 different frequencies, 5 different phase disparities and 21 different horizontal disparities, so the population *C*(*θ*, *f*,Δφ,Δ*x*_enc_) consists of 3150 different correlation-detectors.

Normalizing the stereo energy *E* so as to obtain the effective binocular correlation *C* removes the confounding effect of monocular contrast, making it much easier to extract the stimulus disparity from peaks in the population activity. *C* has the useful property that it is exactly equal to 1 when the stimulus disparity matches the cell's preferred disparity. This is true for *any* pair of stereo images, irrespective of spectral content etc, provided only that the left eye's image is related to the right eye's image by exactly the same offset relating left and right receptive fields. Under these circumstances, *v*_L_(*θ, f,φ,Δφ,Δx*_enc_) = *v*_R_(*θ, f,φ,Δφ,Δx*_enc_) for all *θ, f,φ,Δφ,Δx*_enc_; 2*v*_L_ *v*_R_ is then the same as *v*_L_^2^+*v*_R_^2^, and it follows immediately that *C* = 1.

#### Noise

As Figure 2 makes clear, these neurons become effectively tuned to non-zero vertical disparities only when stimulated at their non-optimal horizontal disparity. Thus, in this model, vertical disparity is encoded only by neurons firing at below their optimal rate. Given this, it becomes important to be sure that this signal would not be lost in noise in a real neuronal population. To incorporate realistic neuronal noise, I convert the correlation *C*, which can take values [−1,1], into an observed spike count, which is necessarily positive or zero. First, I define the mean spike count, *R*_m_, as *R*_m_ = *U*(1+*C*), where *U* is the mean number of spikes elicited by a binocularly uncorrelated stimulus. R_m_ is in the range [0,2*U*], where 2*U* is the mean number of spikes a perfectly binocularly correlated stimulus elicits from neurons tuned to its disparity. I model neuronal noise as a Poisson process [32], [33]. Thus, the actual number of spikes elicited by the stimulus on any given presentation is *R*, where *R* is a random variable drawn from a Poisson distribution with mean *R*_m_.

The effective level of neuronal noise then depends on the value chosen for *U*. This will depend on the neurons' maximal firing rate and the length of time assumed to be available for the judgment. If we assume that the firing rate for the optimal disparity is 100Hz [34] and that the neuronal response is averaged over a 160ms window (since humans can discriminate temporal changes in disparity up to ∼6Hz, [35]), this suggests that the most active neurons might fire 16 spikes in the time available for a disparity judgment, yielding an estimate of around 8 spikes for *U*. Since the variance of Poisson noise is equal to its mean, larger values of *U* produce lower noise, and smaller values would mean greater neuronal noise. In fact, as I discuss below, the model is extremely resilient to neuronal noise. To demonstrate this, the results presented here use *U* = 1. This means that the average neuron fires only 1 spike in the time available for a perceptual judgment, resulting in a very large amount of neuronal noise (coefficient of variance 70% for even optimally-tuned neurons).

Variation in the stimuli also contributes an additional effective source of noise. In this model, a stereo stimulus where left and right images are related simply by a shift will always produce an effective binocular correlation of *C* = 1 in neurons tuned to the disparity of the stimulus. However, neurons which are not tuned to the stimulus will produce a correlation which is on average less than 1, but whose precise value depends on the particular properties of the image, e.g. where the regions of high and low contrast happen to fall in relation to the receptive fields. When it comes to estimating the disparity of a single image, this stimulus-driven variation in response has the same deleterious effect as neuronal noise. If the stimulus disparity has a vertical component, it will stimulate none of the neurons optimally, meaning that *C* will be less than 1 (thus variable) for all neurons, and the neurons will be firing at a lower rate (thus subject to more Poisson noise). Thus, both sources of noise are larger for stimuli with vertical disparity.

### Disparity decoding

#### Storing templates

**Figure 4:**
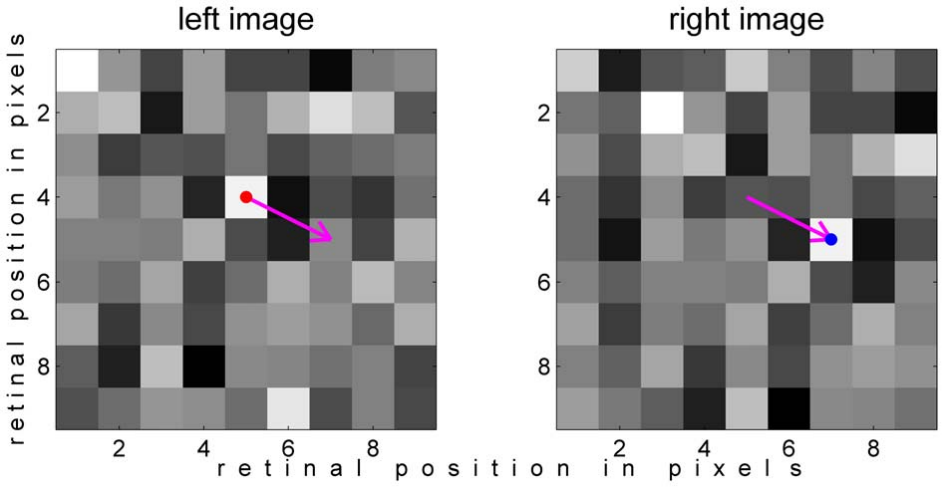
Example image-pair. These have horizontal disparity 2 pixels and vertical disparity 1 pixel. For clarity, these images are just 9×9 pixels; the actual images used in the simulations were 81×81 pixels. The colored dot marks corresponding pixels in the left and right images; the pink arrow shows the disparity vector. Matlab code to generate this figure is Protocol S2.

The first step was to generate many examples of the population's response to stimuli of known disparity. These “template” stimuli were uniform-disparity random noise patterns. Each pixel in the left eye's image, *I*_L_, was given a random value drawn from a Gaussian with zero mean and unit standard deviation. The right eye's image, *I*_R_, was offset horizontally and/or vertically from the first eye's image, and new random pixels were generated to fill the gap (Figure 4).

I produced random noise images with different horizontal and vertical disparities Δ*x*_stim_ and Δ*y*_stim_. Δ*x*_stim_ and Δ*y*_stim_ both ranged from −10 to 10 pixel in steps of 1 pixel, making a total of 441 different two-dimensional stimulus disparities. At each of these 441 stimulus disparities, I generated 500 random image-pairs, each generated with a different random seed *j*, making a total of 220,500 test stereograms.

For each image-pair (Δ*x*_stim_,Δ*y*_stim_, *j*), I calculated the effective binocular correlation as described in Equation 4. I converted this to a mean spike count, and averaged this over 500 different random images, to obtain
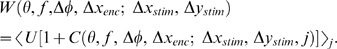
(5)
*W* is the mean number of spikes produced by sensors tuned to orientation θ, frequency *f*, phase disparity Δφ and horizontal disparity tuning Δ*x*_enc_, when averaged over many different presentations of many different noise images with the same 2D stimulus disparity (Δ*x*_stim_,Δ*y*_stim_). The averaging over different presentations of the same image removes the neuronal noise, while the averaging over different images removes stimulus-dependent noise. I envisage this as representing the information stored in the system as a result of visual experience.

#### Template matching

The disparity of an unknown test stimulus can then be estimated by comparing the response of the population to that particular test image with the stored, average response elicited by stimuli with known two-dimensional disparity. The stimulus is taken to have the 2D disparity whose stored activity profile best matches the current activity [21].

Let *R*_test_(*θ*, *f*, Δ*φ*, Δ*x_enc_*) be the number of spikes fired by the encoding population to the particular test image under consideration. Remember that this neuronal population includes cells tuned to 6 different orientations θ, 5 different frequencies *f*, 5 different phase disparities and 21 different horizontal disparities Δ*x*_enc_, so *R*_test_(*θ*, *f*, Δ*φ*, Δ*x*_enc_) is a set of 3150 individual spike-counts. To estimate the disparity of the test stimulus, I compare the population's response to the test image, *R*_test_(θ, *f*, Δ*φ*, Δ*x_enc_*), with the stored mean spike-counts, W, for each of the 441 template stimulus disparities. That is, for each possible two-dimensional disparity (Δ*x*_dec_, Δ*y*_dec_) (subscript “dec” for decoding), I calculate the Pearson correlation coefficient, *r*(Δ*x*_dec_, Δ*y*_dec_), between the set of 3150 spike-counts obtained for this particular test image, *R*_test_(θ, *f*, Δφ, Δ*x_enc_*), and the set of 3150 values stored in *W*(*θ*, *f*, Δ*φ*,Δ*x*_enc_;Δ*x*_dec_, Δ*y*_dec_):
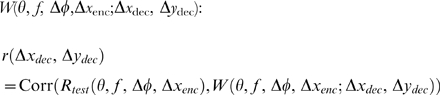
where Corr(a,b) represents the usual Pearson product-moment correlation coefficient between a and b:
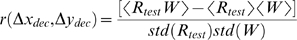
(6)where the sum Σ, averages <> and standard deviations std are all taken over *θ*, *f*, Δ*φ*, Δ*x*_enc_, while holding Δ*x*_dec_ and Δ*y*_dec_ constant.

I shall always use the word Pearson when referring to this correlation, in order to avoid possible confusion with the effective binocular correlation computed by the encoding neurons, Equation 4. In the figures, I shall use a “jet” colormap (running from blue-green-red) to represent spike-counts based on effective binocular correlation, and a “hot” colormap (black-red-yellow-white) to represent Pearson correlation.

To model the lack of sensitivity to disparity in anti-correlated stereograms [36], [37], [38], [39], [40], I finally set any negative correlations to zero, computing
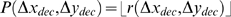
(7)where ⌊⌋ indicates halfwave rectification: ⌊*x*⌋ = *x* for *x*>0, and zero otherwise.

The two-dimensional disparity of the test stimulus is then taken to be the values (Δ*x*_dec_, Δ*y*_dec_) which maximizes the halfwave-rectified Pearson correlation *P*(Δ*x*_dec_,Δ*y*_dec_).

Matlab code (The Mathworks, Natick, MA; www.mathworks.com) to run the simulations and generate most of the figures is available as Supplementary Material (although due to the size of the neuronal populations, running all the simulations presented in this paper takes weeks). Details of which functions to use are given in each figure legend. Other functions called by this code are grouped together in the file Protocol S11.

## Results

### All members of the neuronal population are tuned to zero vertical disparity

**Figure 5:**
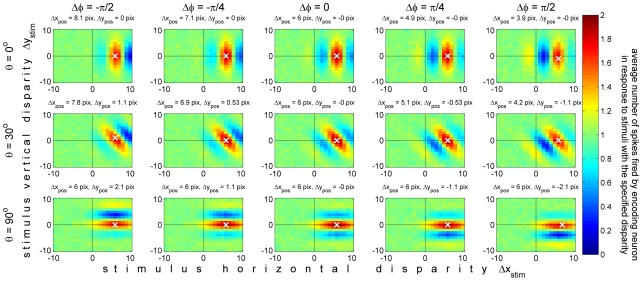
Disparity tuning surfaces for 15 example disparity-encoding neurons with different phase disparities and orientations. Each panel represents the 2D disparity tuning surface for one neuron, that is, the mean spike count elicited from that neuron in response to stimuli with the two-dimensional disparity specified on the horizontal and vertical axes. Specifically, each panel shows W(θ,f,Δφ,Δx_enc_;Δx_stim_,Δy_stim_) (Equation 5), as a function of Δx_stim_ and Δy_stim_, for Δx_enc_ = 6pix, spatial frequency tuning *f* = 0.071cyc/pix, and the different θ and Δφ specified in the row/column labels. Each neuron's two-dimensional position disparity (Δx_pos_,Δy_pos_) is indicated at the top of each panel. This was set as in Equation 1, to ensure its preferred horizontal disparity is Δx_enc_ (here 6pix) and its preferred vertical disparity is 0. The white cross marks the pixel for which the spike count was highest. The fact that this empirical preferred disparity closely agrees with the desired value (6,0) shows that the position disparity successfully cancels out any vertical component introduced by the phase disparity. Matlab code: The mean response was obtained with Protocol S3, averaging over 500 stimuli, and the figure was generated with Protocol S4.

First, it is important to establish that – despite their wide range in phase disparity, position disparity and orientation – all the units in our encoding population genuinely are tuned to zero vertical disparity. To this end, Figure 5 shows two-dimensional disparity tuning surfaces for 15 example members of the model population of 3150 neurons. Disparity tuning surfaces like this have been measured for real neurons by Cumming [13], Durand et al, [10], [11] and Gonzalez et al [12]. Each panel in Figure 5 shows the disparity tuning surface for a different model neuron in the encoding population. The pseudocolor represents the mean number of spikes fired by that neuron to stimuli with a given disparity, averaged over many different random noise images. All the neurons shown have the same spatial frequency, f = 0.071cyc/pix, and preferred horizontal disparity, Δ*x*_enc_ = 6pix. The three rows show neurons tuned to different orientations: vertical, oblique and horizontal, as specified to the left of each row. The five columns show neurons with different phase-disparities Δ*φ*, as labelled at the top of each column. The phase disparity controls the symmetry of the disparity tuning surface: odd-symmetric for Δ*φ* = ±*π*/2, even-symmetric for Δ*φ* = 0, intermediate for Δ*φ* = ±*π*/4. As described in the Methods, phase disparity shifts the preferred disparity in a direction orthogonal to the neuron's orientation. Model neurons in the encoding population were given just the right amount of position disparity (Equation 1) to cancel this out and place their peak sensitivity in the region expected for normal vision. This 2D position disparity (Δ*x*_pos_,Δ*y*_pos_) is indicated above each panel. When there is no phase disparity (Δ*φ* = 0, middle column), the position disparity is simply equal to the desired disparity tuning, here (6,0). Elsewhere, the model neurons have to be given additional amounts of vertical and/or horizontal position disparity in order to bring the preferred 2D disparity back to the desired value. The white cross in each panel marks the stimulus disparity which elicited the highest response from that neuron, averaged over the 500 images. In every case this is very close to (6,0), indicating that the position disparity specified in Equation 1 has had the desired effect. This was true for all 1350 neurons in our population, as well as the 15 examples shown in Figure 5, demonstrating that Equation 1 achieves its aim of making all neurons in the encoding population respond best to zero vertical disparity.

### Vertical disparity is implicitly encoded in the pattern of activity across the population

**Figure 6:**
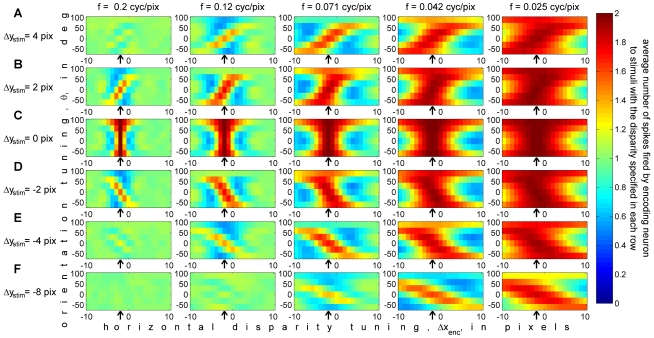
Average population response, W(θ,f,Δφ,Δx_enc_;Δx_stim_,Δy_stim_), for different stimulus vertical disparities. Only neurons with zero phase disparity are shown (the key features discussed in the text are the same for all phase disparities). The stimulus disparity is fixed in each panel, and the horizontal axis is the preferred horizontal disparity of the neurons (unlike Figure 5, where the neuron's preferred horizontal disparity was fixed in each panel and the horizontal axis was the horizontal disparity of the stimulus). Each panel shows the mean number of spikes which stimuli with this disparity elicit from 126 neurons, tuned to 21 different horizontal disparities Δx_enc_ and 6 orientations θ, plotted on the horizontal and vertical axes respectively. The 5 panels in each row show sets of 126 neurons tuned to 5 different preferred spatial frequencies. Thus together each row shows the mean response of the zero-phase-disparity sub-population, 630 neurons, averaged over 500 random stimuli with the same stimulus disparity. The stimulus horizontal disparity, Δx_stim_, was set equal to −2 pixels throughout (marked with the arrow in each panel); the stimulus vertical disparity, Δy_stim_, was set to a different value in each row, as indicated to the left of each row. The colorscale is the same as in Figure 5, indicated on the right. Matlab code: The mean responses were obtained with Protocol S3, and the figure was generated with Protocol S5.

We now move to considering how stimulus vertical disparity is encoded within this population. To do this, instead of plotting the mean response of individual neurons to stimuli with different disparities, as was done in Figure 5, we now plot the mean response of many neurons to stimuli with a given disparity. This is what is shown in Figure 6.

Each row of Figure 6 shows the average spike count, *W*(*θ*, *f*,Δ*φ*,Δ*x*_enc_;Δ*x*_stim_,Δ*y*_stim_), for all zero-phase-disparity neurons in the population, elicited by one particular stimulus disparity (Δ*x*_stim_,Δ*y*_stim_). (The choice to display the 630 neurons with Δφ = 0 is arbitrary; qualitatively similar plots are obtained for the other phase disparities.) The 6 rows show the response of this population to 6 different stimulus vertical disparities Δy_stim_, as indicated to the left of each row. In each case the stimulus horizontal disparity is Δ*x*_stim_ = −2 pixels, marked with the arrow in each panel. Each panel shows *W*(*θ*, *f*,Δ*φ*,Δ*x*_enc_;Δ*x*_stim_,Δ*y*_stim_) as a function of Δ*x*_enc_ (horizontal axis) and θ (vertical axis), for the spatial frequency *f* indicated at the top of the column. Thus, the 6 rows of Figure 6 correspond to 6 of the 441 stored responses of this population, which will be used in our template-matching algorithm to extract an estimate of stimulus disparity.

The neurons above the arrow in each panel are those tuned to the horizontal disparity of the stimulus under consideration, Δ*x*_enc_ = Δ*x*_stim_. As one would expect, the effective correlation is generally high in this region (dark red colors). The stimulus vertical disparity Δ*y*_stim_ is 4 pixels in row A, 2 pixels in row B, 0 pixels in row C, and so on as indicated to the left of each row. Although the cells in the population are tuned to many different horizontal disparities, Δ*x*_enc_, they are all tuned to zero vertical disparity. Thus the middle row, Figure 6C, is the only case where any neurons are tuned to the exact two-dimensional disparity of the stimulus. Here, neurons with Δ*x*_enc_ = Δ*x*_stim_ = −2 have receptive fields which exactly match the binocular disparity of the stimulus. Their correlation is therefore *C* = 1 for every noise image with this disparity, and so the mean spike-count *W* = (1+*C*) is exactly 2. The mean spike-count falls below 2 to either side of the arrow, as the difference between the horizontal disparity of the stimulus and that preferred by the neurons increases. The rate of decrease depends on the spatial frequency channel, since this controls the size of the receptive fields. For the left-most column, *f* = 0.2 cycles/pixel, the standard deviation of the receptive field envelope, σ, is just 1.25 pixels. For the right-most column, *f* = 0.025 cycles/pixel and σ = 10 pixels, meaning that the effective correlation experienced by these neurons is still high even for neurons tuned to disparities several pixels away from the stimulus. The rate of decrease also depends on the orientation. In our model population, the receptive field envelopes are isotropic, but the rate of change of the receptive field function is still fastest orthogonal to the cell's preferred orientation *θ* (see Figure 3). Thus, for each spatial frequency channel, the rate of change along the horizontal direction is fastest for the vertically-oriented cells (*θ* = 0°), and slowest for the horizontally-oriented ones (*θ* = ±90°). This effect can be seen in Figure 6C: the red region of high correlation extends further to either side of the optimal disparity for the horizontally-oriented cells at the top and bottom of each panel.

The same effect of receptive-field size can be seen as we look at rows other than row C, thus increasing the distance between the neurons' preferred vertical disparity (0) and that of the stimulus. The peak response anywhere in the population declines as we move along a column away from Δ*y*_stim_ = 0, as described by Read & Cumming [15]. Again, this decrease is most apparent for the higher-frequency channels, where receptive fields are smaller. For the highest-frequency channel (0.2 cyc/pix), where σ is just 1.25 pixels, a vertical disparity of −8 pixels (row F) is enough to make the portions of the images falling within the left and right-eye receptive fields completely uncorrelated. This means that the average binocular correlation is zero, and so with the spiking model I have adopted, the mean spike count is just 1, everywhere in the panel.

The most interesting, and informative, panels of Figure 6 are those where the stimulus has a non-zero, but relatively small, vertical disparity (rows A,B,D,E). Here, the effective binocular correlation C has fallen below 1, but is still above zero. In this case, the red region of high spike-counts takes on a distinctive diagonal slant, whose direction depends on the sign of stimulus vertical disparity. Where stimulus vertical disparity is positive (rows A, B), spike-counts are highest for receptive fields tilted counter-clockwise from vertical (positive *θ*) when horizontal disparity is positive, and for receptive fields tilted clockwise from vertical (negative *θ*) when horizontal disparity is negative. When stimulus vertical disparity is negative (rows D, E, F), the situation is reversed. The reason is exactly the geometry sketched in Figure 2. This slant is the “signature” of vertical disparity, and will enable us to decode vertical disparity from this population.

### 2D stimulus disparity can be extracted from the response of this population

Figure 6 showed the average response of a neuronal population, averaged across thousands of stimuli with the same disparity. As we have seen, this average response possesses a structure which reflects the vertical disparity of the stimulus. However, this averaging process conceals important features of the response to single images. Most importantly, the response of the neuronal population to single images is affected not only by the disparity, but also by the luminance features of the particular image. These features cancel out to nothing when averaged over many random images, but the brain cannot take advantage of this when estimating the disparity of a single image. The stereo correspondence problem is complicated by these “false matches” due to particular features of the image [31]. Normalizing stereo energy so as to calculate the effective binocular correlation *C* is enough to solve the problem in the absence of vertical disparity. Then, as explained in the Methods, the stimulus horizontal disparity can be identified from the horizontal disparity tuning of the cell with *C* = 1 (mean spike count = 2*U*). However, when there is a mismatch between the cell's preferred vertical disparity and the vertical disparity of the stimulus, the correlation will not usually reach 1 even for cells tuned to the horizontal disparity of the stimulus, so the false-match problem creeps in again. Secondly, neuronal populations are subject to noise. In principle, this may be reduced by averaging either over a long time period, or over a large pool of neurons with similar tuning and independent noise. Here, I have made the conservative assumption that neither of these options is available, so the neuronal population is subject to very large amounts of trial-to-trial noise, with the coefficient of variation at least 70%.

**Figure 7:**
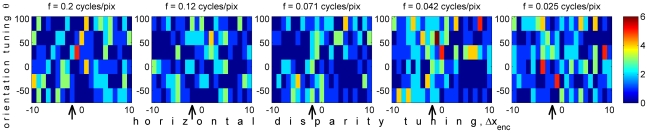
Neuronal spike counts, R_test_(θ,f,Δφ,Δx_enc_), elicited by a single presentation of a single test image, with stimulus disparity (Δx_stim_, Δy_stim_) = (−2, +2). As in Figure 6, only neurons with zero phase disparity are shown, Δφ = 0. The different panels each show 126 neurons tuned to different spatial frequencies *f*, while 21 preferred horizontal disparity tunings Δx_enc_ and 6 orientations θ are shown by the horizontal and vertical axes, respectively. In each panel, an arrow marks the neurons tuned to the horizontal disparity of the stimulus. The colorscale is the same in all panels. The average response of the population to all Gaussian-noise stimuli with this disparity was shown in Figure 6B (note different colorscale). This mean response differs from the single-stimulus response shown here because the latter is affected by stimulus-dependent variation, reflecting the properties of this particular image, and Poissonian noise on neuronal spiking. Matlab code: This figure was generated by Protocol S6.

To bring home just how much variation these two sources of noise contribute, Figure 7 shows the spikes elicited in response to a single example test image, with stimulus disparity Δ*x*_stim_ = −2 and Δ*y*_stim_ = +2 pixels. For comparison, Figure 6B showed the average response of the same population to stimuli with this disparity, with both neuronal and stimulus-driven noise averaged away. The 5 panels of Figure 6B are thus the “template” which Figure 7 is meant to match (though note that because up to 6 spikes were produced by the single presentation in Figure 7, while the mean number of spikes never rises above 2, different colorscales were used in the two plots). At first glance, the task might appear to be impossible, given the very high levels of noise. However, certain features of similarity are indeed detectable between Figure 7 and Figure 6B. At the lower spatial frequencies (right-hand panels), where the stimulus vertical disparity is not so large as a fraction of receptive field size, there is a slight tendency for neurons tuned to the horizontal disparity of the stimulus, marked with the arrows, to fire more spikes. Similarly, the slanted structure of the most responsive region is already hinted at. Furthermore, recall that for reasons of space, Figures 6 and 7 show only the 630 neurons with zero phase-disparity; once we include the other phase disparities, there are a further 2520 neurons whose instantaneous response can be matched to the corresponding template. As I show below, despite the major differences between the single-image response shown in Figure 7 and its template shown in Figure 6B, the population provides enough information for the correct template to be reliably identified.

**Figure 8:**
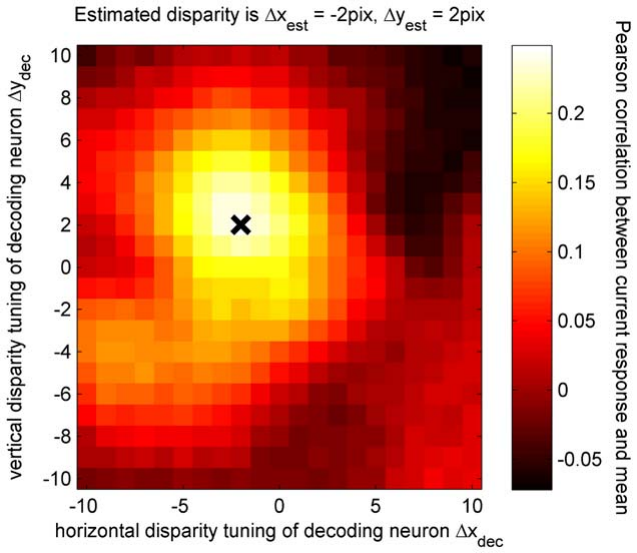
Response of the population of disparity decoders (before rectification) to a test image with horizontal disparity Δx_test_ = −2pix, Δy_test_ = +2pix, marked with the cross. Each pixel in the plot represents a decoding neuron, tuned to the 2D disparity (Δx_dec_,Δy_dec_) indicated on the horizontal and vertical axes. The pseudocolor represents the Pearson correlation coefficient between the activity in the encoding population elicited by the test image, and the stored “templates” representing the mean activity to stimuli with disparity (Δx_dec_,Δy_dec_). The disparity of the test image was correctly estimated from the peak activity in the decoding population. Matlab code: This figure was also generated by Protocol S6.

As described in the Methods, I assess the quality of the match between the population response to a single image and the stored average population response by calculating the Pearson correlation coefficient between the two. Figure 8 uses pseudocolor to show the Pearson correlation coefficients *r*(Δ*x*_dec_,Δ*y*_dec_) for all 441 disparities. The black cross marks the disparity of the stimulus. In this example, the highest Pearson correlation is obtained from the decoder tuned to this disparity, so for this single test image, the stimulus disparity is correctly extracted.

**Figure 9:**
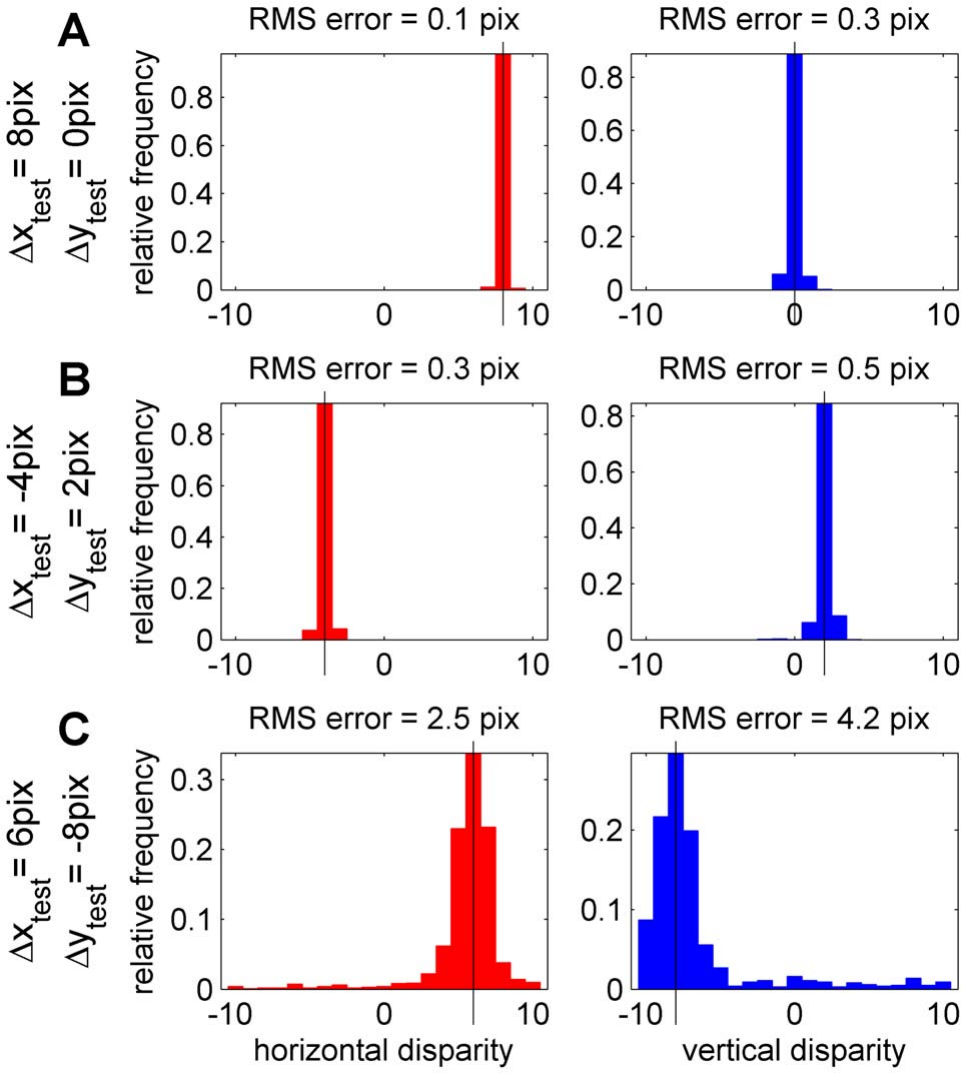
Results of estimating 2D stimulus disparity from the 1D disparity encoding population. Each panel shows the distribution of the estimated disparity component (left column, red: horizontal disparity; right column, blue: vertical disparity). The rows show three different test disparities (Δx_test_,Δy_test_), as indicated by the black vertical lines in each column. In each case, 1000 images with the specified test disparity were generated, and their 2D disparity was estimated as being the value of (Δx_dec_,Δy_dec_) which gave the best match between the population activity R_test_(θ,f,Δφ, Δx_enc_) evoked by the test image, and the stored W(θ,f,Δφ,Δx_enc_;Δx_dec_,Δy_dec_), as in Figure 8. The root-mean-squared error between the estimated disparity and the correct value is indicated at the top of each panel. Matlab code: The disparity estimates were obtained with Protocol S7, and the figure was generated with Protocol S8.

Figure 9 quantifies the accuracy with which this algorithm performs across many test images. The plots show frequency histograms for the estimated disparity (red for horizontal disparity, blue for vertical) for 1000 different random test images with a fixed disparity. None of the 1000 test images was in the set of 500 images used to obtain the template responses, although they were all Gaussian noise images like those in Figure 4. Each column in Figure 9 shows results for a different test disparity (Δ*x*_test_,Δ*y*_test_). The root-mean-squared error between the disparity estimated for each test image and its actual value is given above each panel. The algorithm's performance does not depend on the horizontal disparity of the test image (provided, of course, that it falls within the range to which the encoding population is tuned), so the three particular horizontal disparities chosen are immaterial. In contrast, performance does depend strongly on the vertical disparity tested. The three rows of Figure 9 show results for increasing vertical disparity magnitudes: A: Δ*y*_test_ = 0, B: Δ*y*_test_ = 2, C: Δ*y*_test_ = −4 pixels.

In Figure 9A, the test images had zero vertical disparity. Thus, the encoding population contains sensors tuned to the exact 2D disparity of the test images. Under these circumstances, unsurprisingly, both horizontal and vertical disparity are reconstructed with great accuracy. In Figure 9B, the test images had a vertical disparity of 2 pixels. An example population response to a single test image with this disparity was shown in Figure 7, while the template response (averaged over many training images with this disparity) was shown in Figure 6B. Here, no sensors in the encoding population are tuned to the 2D disparity of the stimulus. This naturally reduces the accuracy, but the RMS error is still only half a pixel. Critically, both the magnitude and sign of the vertical disparity can still be estimated from the reduction in the peak spike count [15] and the slant in the region of high spike count.

Figure 9C shows results when the test images had a vertical disparity of −8 pixels. This is large compared to the receptive field size of most channels, so the RMS error increases further, but the sign of the vertical disparity is still reliably detected. Horizontal disparity is also extracted, but with a larger error which would correspond to a reduced stereoacuity. This is qualitatively consistent with human performance: human stereo perception becomes worse as vertical disparity increases, and is destroyed by relatively small amounts [41], [42]. Here, almost all the “work” is being done by the low spatial-frequency channels, but these are still enough to extract 2D disparity, without being excessively degraded by the higher-frequency channels for which the stimulus is effectively uncorrelated. Ultimately, of course, as vertical disparity moves beyond the range spanned by the largest receptive fields, performance will fall to chance, again as human performance does.

### Response to anti-correlated stereograms

Disparity is encoded within this model by the population of binocular correlation detectors *C*(*θ*, *f*,Δ*x*). This population, which is all tuned to zero vertical disparity on the retina, performs the initial encoding of disparity. It was chosen to resemble primary visual cortex, V1. For example, these initial disparity encoders are tuned to a particular spatial frequency and orientation, and they continue to respond to disparity in anti-correlated stimuli. Anti-correlated stereograms are those in which one eye's image has been contrast-inverted, so that black pixels are replaced with white. Since I use zero to represent the mean luminance, this corresponds to inverting the sign of one eye's image. Thus, the product *v*_L_ *v*_R_ changes sign when the stimulus is made anti-correlated. This means that the disparity tuning of binocular correlation-detectors inverts for anti-correlated stimuli. A similar inversion is found in V1 [43], [44], although with a slight reduction in amplitude.

Disparity is extracted from the activity of these V1 correlation-detectors by a higher-level brain area. The properties of this decoding area should ideally match those of human perception. For example, neurons in this region should not respond to disparity in anti-correlated stereograms, since these produce no perception of depth in humans or monkeys [36], [37], [40], and neurons in higher visual areas such as IT and V4 do not respond to disparity in anti-correlated stimuli [38], [39]. In this paper, I have used the Pearson correlation coefficient, *r*, to quantify how well the population response to a test image matches the mean population response to template images. To match the lack of response to disparity in anti-correlated stereograms, I set the response of the decoding population equal to the half-wave-rectified Pearson correlation, replacing negative *r* with 0. This has no effect on correlated stereograms, where the maximum *r* is positive, but it prevents the decoder responding systematically to disparity in anti-correlated stereograms.

**Figure 10:**
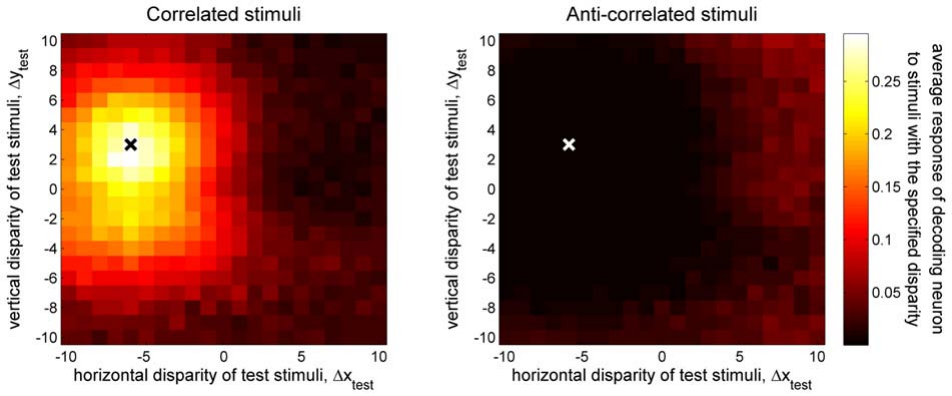
Disparity tuning surface for the disparity decoder tuned to Δx_stim_ = −6 and Δy_stim_ = 3, indicated by the cross in each panel. The color of each pixel in the plot shows the mean response, <P(Δx_stim_,Δy_stim_)>, averaged over 40 test stimuli with the disparity (Δx_test_,Δy_test_) specified by that pixel's position on the horizontal and vertical axes. A: for correlated stimuli. B: for anti-correlated stimuli. The same colorscale is used in both panels. Matlab code: The results were generated by Protocol S9 and the figure was plotted by Protocol S10.

**Figure 11:**
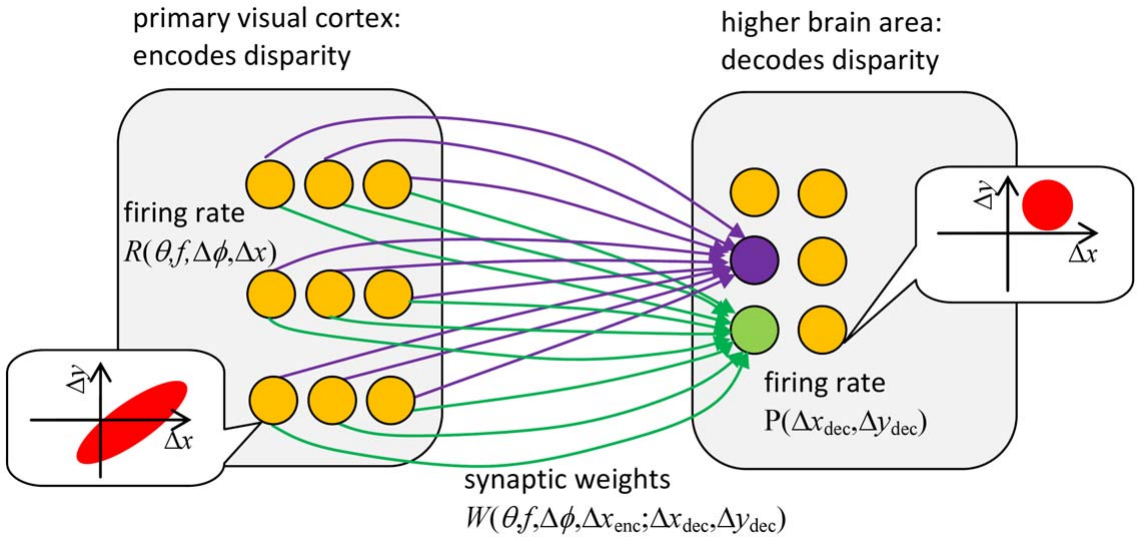
Sketch of the model's physiological interpretation. Disparity is initially encoded by a population tuned entirely to zero vertical disparity. A higher brain area extracts two-dimensional disparity from the activity of this population. The synaptic weights of the projection from the encoding to the decoding population store the mean activity of the encoding population to stimuli with different 2D disparity. For simplicity, synaptic connections onto only two, color-coded, decoding neurons are shown. The call-outs show examples of the 2D disparity tuning for the two populations (encoding: oriented, optimal vertical disparity is zero; decoding: isotropic, optimal vertical disparity may be non-zero).

Figure 8 illustrated the response of the population of disparity decoders (prior to the half-wave rectification) to one example test stimulus, showing that the maximally-responding decoders were those tuned to disparities close to that of the stimulus. Figure 10 plots the disparity tuning surface of a single disparity decoder, the one tuned to (Δ*x*_stim_,Δ*y*_stim_) = (−6,−3), for both correlated and anti-correlated stereograms. The pseudocolor of each pixel shows the mean <*P*(Δ*x*_stim_,Δ*y*_stim_)> averaged across 40 different random images with the same disparity (Δ*x*_test_,Δ*y*_test_), specified by the pixel's position on the axes. Figure 10A shows the disparity tuning surface for normal, correlated stereograms. Unsurprisingly, the response is largest when the two-dimensional disparity of the test stimulus matches the preferred disparity of the decoder, indicated with the cross. Similar disparity tuning surfaces were plotted in Figure 5 for the encoding neurons. The disparity tuning surfaces for the decoding neurons differ in two respects. First, they are isotropic rather than elongated, because the decoding neurons receive inputs from cells tuned to all orientations (Figure 11). Second, the peak response is obtained for a non-zero vertical disparity, whereas the encoding neurons were all tuned to zero vertical disparity.

Figure 10B shows the disparity tuning surface for the same decoder as in Figure 10A, but this time obtained with anti-correlated stereograms. As noted, anti-correlated stimuli elicit no perception of depth, and neurons in brain areas which are believed to have solved the correspondence problem do not discriminate disparity in anti-correlated stereograms. The Pearson correlation coefficient *r* between the response to an anti-correlated stereogram and the stored average responses for correlated stereograms is almost always negative, meaning that half-wave rectification ensures the decoder response *P*(Δ*x*_stim_,Δ*y*_stim_) is zero. Accordingly, the disparity tuning surface in Figure 10B is almost completely flat, in agreement with the physiological data for areas IT and V4 [38], [39]. Thus, both encoding and decoding neurons in this simulation have properties consistent with those of the corresponding neuronal populations, as far as these are known.

## Discussion

This paper has implemented a simple physiologically-inspired two-dimensional stereo correspondence algorithm. It consists of two model “brain areas”: one which performs the initial encoding of binocular disparity between left and right images, and one which decodes this activity so as to arrive at an estimate of the two-dimensional disparity in the images. The unusual feature of this model is that the encoding neurons are all tuned to the same vertical disparity (zero). Despite this, the decoding neurons are able to successfully recover 2D stimulus disparity. This is possible because vertical disparity causes distinctive patterns of activity across the encoding population. The model uses its stored knowledge about these patterns, in the form of templates of expected activity, to deduce the stimulus disparity.

### Neuronal correlates

The model has a simple physiological interpretation. The population of disparity encoders, C(θ,f,Δx_enc_), was designed to represent primary visual cortex, V1. Neurons in this area are tuned to different orientations *θ*, spatial frequencies *f* and horizontal disparities Δ*x*_enc_, and respond to disparity in anti-correlated stereograms. This encoding area projects to a higher brain area which extracts stimulus disparity. Neurons in this decoding area are tuned to both horizontal and vertical disparity, but are not sensitive to orientation or spatial frequency. They do not respond to disparity in anti-correlated stereograms. The perceived disparity corresponds to the preferred disparity of the most active neuron in the decoding area.

The stored templates of the population activity expected for different stimulus disparities, *W*, can be viewed as the synaptic weights in the projection from the early encoding area to the decoding area (Figure 11). That is, *W*(*θ*, *f*,Δ*φ*,Δ*x*_enc_;Δ*x*_dec_,Δ*y*_dec_) describes the strength of the synaptic connection from the encoding neuron tuned to orientation *θ*, frequency *f*, phase disparity Δ*φ* and horizontal disparity Δ*x*_enc_, onto the decoding neuron tuned to horizontal disparity Δ*x*_dec_ and vertical disparity Δ*y*_dec_. The firing rate of the decoding neuron depends on the total activity of its input neurons weighted by the strength of each synapse (the term *Σ R*_test_ *W* in Equation 6), after undergoing a subtractive and a divisive normalization, and finally a threshold non-linearity (Equation 7). The threshold non-linearity is a universal feature of neuronal circuits, since firing rates cannot go negative. Both subtractive and divisive normalization have been extensively discussed in the literature, and plausible neuronal mechanisms have been proposed to implement them [45], [46], [47], [48], [49], [50].

### Robustness to noise

This model is able to successfully decode two-dimensional disparity, including both the magnitude and sign of vertical disparity, from the activity of the encoding population. This demonstrates that information regarding vertical disparity is implicitly encoded within this population. The accuracy of this information, unsurprisingly, declines as the vertical disparity of the stimulus increases (Figure 9), consistent with psychophysical data. In the model, this decline occurs because information about the stimulus disparity is being carried by neurons which are not optimally tuned to it. Partly, this is because of neuronal noise: the effective signal-to-noise level declines as we move away from the peak of the neuron's tuning surface. I modelled neuronal spiking as a Poisson process, and deliberately chose a low spike count so that the Poisson noise would be large. In these simulations, neurons optimally tuned to the stimulus disparity have a coefficient of variation (CV, the ratio of standard deviation to mean) of 70%, while neurons which are tuned so far from the stimulus disparity that it appears effectively uncorrelated to them have a CV of 100%. However, the main reason for the decline in decoding accuracy is not neuronal noise, but fluctuations in the stimulus. For the uniform-disparity stimuli examined here, receptive fields tuned to the 2D stimulus disparity always experience an effective binocular correlation of exactly 1 (CV = 0%), whereas away from the 2D stimulus disparity the effective binocular correlation is, on average, smaller, and also much more variable. This means that as vertical disparity moves away from the value to which the neurons are tuned (here, zero), the stimulus-dependent fluctuations contribute much more variability to the neuronal spiking.

Nevertheless, despite these two potent sources of noise in the model, the simulations reveal that it performs extremely well. This is because the decoding process uses the responses of thousands of encoding neurons. Although every neuron is tuned to different parameters, and so their responses cannot be directly pooled, the decoding process effectively averages out noise when it correlates the responses of thousands of neurons with the stored templates. For this reason, the model is extremely robust to neuronal noise. If the reader runs the code in the Supplementary Material, reducing the Poisson noise by setting Neurons.MeanSpikeUncorr to a value greater than its current value of 1, s/he will be able to verify that the results show only a slight improvement in accuracy.

### Relationship to previous models of vertical disparity encoding

The model of Read & Cumming [15] was discussed in the Introduction. That model worked by detecting changes in vertical disparity magnitude across the visual field. In contrast, the present model is purely local; all neurons simulated were tuned to the same cyclopean position in the visual field. This model would therefore work even with the induced-effect stimulus of Serrano-Pedraza & Read [16]. Serrano-Pedraza & Read [16] were correct to reject the particular decoding model proposed by Read & Cumming [15], but wrong to conclude that vertical disparity must be explicitly encoded. A more sophisticated decoding of the same encoding population is consistent with their psychophysical results.

Matthews et al. [51] also modelled the perceptual effects of vertical disparity using energy-model neurons with different orientation tuning. The present algorithm differs substantially from theirs. Most importantly, their model does not ever estimate stimulus vertical disparity. Their decoding algorithm extracts a one-dimensional estimate of horizontal disparity, assuming that vertical disparity is zero. This means that when vertical disparity actually is present, it causes horizontal disparity to be mis-estimated: a vertical disparity V is misinterpreted as a horizontal disparity of Vcotθ, where θ is the cell's preferred orientation relative to horizontal (eq. 6 of Matthews et al.). They postulate that the perceptual effects of vertical disparity are a direct consequence of this confusion between horizontal and vertical disparity components. In contrast, the present model explicitly decodes both horizontal and vertical disparity. Vertical disparity does not cause horizontal disparity to be systematically mis-estimated (although it does increase the random error, Figure 9). Thus, the present model is agnostic on the question of how vertical disparity causes its perceptual effects: the two-dimensional disparity decoded by the present algorithm would have to be fed into one of the many models of that process (e.g. [14], [52],[53],[54],[55]. Second, in order to explain how the “mistaken” disparity *V*cot*θ* produces a perceptual effect when averaged over neurons tuned to all possible orientations *θ*, Matthews et al. [51] invoke a radial bias for *θ* [56], [57], [58]. The present algorithm does not depend on any such anisotropy. In the simulations presented here, *θ* was assumed to be isotropic; any anisotropy would not affect the performance of the algorithm. This means that the present model is almost the opposite of that in Matthews et al. Their neuronal population explicitly encodes both horizontal and vertical disparity, but their decoding algorithm deliberately extracts only horizontal disparity. My population explicitly encodes only horizontal disparity, but my decoding algorithm extracts both horizontal and vertical disparity.

### Consistency with known physiology

As sketched in Figure 2, the present algorithm depends critically on the obliquely-oriented disparity-tuning surfaces predicted by the stereo energy model. It is therefore important to know whether real neurons display such oriented disparity-tuning surfaces. In monkey V1, Cumming [13] examined two-dimensional disparity-tuning surfaces for random-dot patterns, and compared their orientation to the cell's orientation tuning for grating stimuli. He found many cells with the obliquely-oriented disparity tuning used here. However, most cells had disparity-tuning surfaces elongated along the horizontal axis, independent of the cell's orientation tuning for gratings. Cumming argued that this represented a specialization for horizontal disparity not predicted by the energy model. This non-energy-model population can be modeled by combining several energy-model units with different horizontal disparity tuning [3]. The oblique disparity tuning predicted by the energy model is also found in cat visual cortex [59], and in peripheral monkey V1 [11]. Thus, the existing physiological evidence suggests that neurons with the obliquely-oriented disparity-tuning surfaces used by this model do exist, and may form the inputs for a second stage of disparity encoding consisting of neurons with horizontally-oriented disparity-tuning surfaces.

Neurons in V1 contain both position and phase disparity [22], [23], [24], [60]. The model presented here works equally well whether position disparity alone, or both position and phase disparity, are included. In this paper, I specified a relationship between position disparity, phase disparity, frequency and orientation (Equation 1) which ensured that all neurons in the population were tuned to zero vertical disparity. (If this relationship did not hold, the model would contain neurons tuned to a range of vertical disparities, so its success would be trivial.) No physiological study has yet quantified both phase disparity and vertical disparity tuning, yet the results of [13] imply that something like Equation 1 may hold in reality, at least in the central 10° or so of the visual field.

In the visual periphery, very little is currently known about the distribution of 2D retinal disparity, despite the fact that this is where the range of naturally-occurring vertical disparities is largest [14], [61]. The existing physiological studies have reported their results only in head-centric Helmholtz coordinates, and have not examined tuning as a function of position on the retina. The encoding population described here, where all neurons at a given retinotopic location are tuned to the same vertical disparity on the retina (Figure 1), is consistent with the very limited existing physiological data available [15]. Only future physiological studies can resolve the issue. These should obtain a full 2D disparity tuning surface for every neuron; as Figure 2 shows, 1D cross-sections can give misleading results. They should be clear about the definition of vertical disparity they are using, reporting data in retinal, as well as head-centric, coordinates. Finally, they need to examine disparity tuning as a function of position on the retina (not just eccentricity), in order to test whether the mean and variation in preferred vertical disparity varies across the retina as predicted from natural image statistics [14]. These studies should be carried out in both early visual cortex and in higher areas such as IT believed to underlie perception. The present model predicts that the range of preferred vertical disparities will be larger in the higher cortical areas.

### Significance

This paper demonstrates a highly efficient strategy for representing 2D stimulus disparity. 2D disparity is represented explicitly only at the decoding level, with the initial encoding being one-dimensional. Because the disparity decoding area does not represent other stimulus properties such as orientation, spatial frequency and phase, this results in a huge reduction in the number of neurons required.

Irrespective of whether the model here is ultimately validated physiologically, it nevertheless provides a vivid demonstration that populations of disparity-tuned neurons contain a much richer array of information than previously appreciated. It places a caveat on the common wisdom that in order to encode a quantity *X*, a neuronal population needs to be tuned to a range of values of *X*. In this example, horizontal and vertical disparity are completely independent quantities in the external world, but they are bound together with orientation at the initial encoding stage in the brain. Subsequently, vertical disparity can be extracted from neurons via their tuning to horizontal disparity and orientation alone. Under these very special circumstances, the common wisdom ceases to hold.

## Supporting Information

Protocol S1 Matlab code for running the simulations presented in this paper (Fig_ExampleRFs.m)
(1.00 KB TXT)

Protocol S2 Matlab code for generating Fig 4 (Fig_ExampleImages.m)
(1.00 KB TXT)

Protocol S3 Matlab code (www.mathworks.com) for running the simulations presented in this paper (gets templates). GetTemplates.m
(1.00 KB TXT)

Protocol S4 Matlab file, for generating Fig 5 (Fig_DispTunSurfEncoders.m)
(3.00 KB TXT)

Protocol S5 Matlab code (www.mathworks.com) for running the simulations presented in this paper (Fig_MeanResponses.m)
(2.00 KB TXT)

Protocol S6 Matlab code (www.mathworks.com) for running the simulations presented in this paper (Fig_FitDisparity.m)
(3.00 KB TXT)

Protocol S7 Matlab code (www.mathworks.com) for running the simulations presented in this paper (FitDisparity.m)
(2.00 KB TXT)

Protocol S8 Matlab code (www.mathworks.com) for running the simulations presented in this paper (Fig_FreqHists.m)
(3.00 KB TXT)

Protocol S9 Matlab code for generating Fig 10 (DispTunSurfDecoders.m)
(2.00 KB TXT)

Protocol S10 Matlab code for generating Fig 10 (Fig_DispTunSurfDecoders.m)
(1.00 KB TXT)

Protocol S11 Zip archive containing 7 files with Matlab functions necessary to run the simulations and generate the figures in the paper (Protocol_S11.zip)
(0.14 MB ZIP)

## References

[1] Liu Y, Bovik AC, Cormack LK (2008). Disparity statistics in natural scenes.. J Vis.

[2] Hibbard PB (2007). A statistical model of binocular disparity.. Visual Cognition.

[3] Read JCA, Cumming BG (2004). Understanding the cortical specialization for horizontal disparity.. Neural Comput.

[4] Helmholtz Hv (1925). Treatise on physiological optics.

[5] Ogle KN (1952). Space perception and vertical disparity.. J Opt Soc Am.

[6] Farell B (1998). Two-dimensional matches from one-dimensional stimulus components in human stereopsis.. Nature.

[7] Serrano-Pedraza I, Phillipson GP, Read JCA (2009). A specialization for vertical disparity discontinuities.. Journal of Vision in press.

[8] Rogers BJ, Koenderink J (1986). Monocular aniseikonia: a motion parallax analogue of the disparity-induced effect.. Nature.

[9] Kaneko H, Howard IP (1997). Spatial limitation of vertical-size disparity processing.. Vision Res.

[10] Durand JB, Zhu S, Celebrini S, Trotter Y (2002). Neurons in parafoveal areas V1 and V2 encode vertical and horizontal disparities.. J Neurophysiol.

[11] Durand JB, Celebrini S, Trotter Y (2006). Neural Bases of Stereopsis across Visual Field of the Alert Macaque Monkey.. Cereb Cortex.

[12] Gonzalez F, Justo MS, Bermudez MA, Perez R (2003). Sensitivity to horizontal and vertical disparity and orientation preference in areas V1 and V2 of the monkey.. Neuroreport.

[13] Cumming BG (2002). An unexpected specialization for horizontal disparity in primate primary visual cortex.. Nature.

[14] Read JCA, Phillipson GP, Glennerster A (2009). Latitude and longitude vertical disparity.. Journal of Vision in press.

[15] Read JCA, Cumming BG (2006). Does visual perception require vertical disparity detectors?. Journal of Vision.

[16] Serrano-Pedraza I, Read JCA (2009). Stereo vision requires an explicit encoding of vertical disparity.. Journal of Vision.

[17] Ohzawa I, DeAngelis GC, Freeman RD (1990). Stereoscopic depth discrimination in the visual cortex: neurons ideally suited as disparity detectors.. Science.

[18] Ohzawa I (1998). Mechanisms of stereoscopic vision: the disparity energy model.. Curr Opin Neurobiol.

[19] Banks MS, Gepshtein S, Landy MS (2004). Why is spatial stereoresolution so low?. J Neurosci.

[20] Filippini HR, Banks MS (2009). Limits of stereopsis explained by local cross-correlation.. J Vis.

[21] Tsai JJ, Victor JD (2003). Reading a population code: a multi-scale neural model for representing binocular disparity.. Vision Res.

[22] DeAngelis GC, Ohzawa I, Freeman RD (1991). Depth is encoded in the visual cortex by a specialised receptive field structure.. Nature.

[23] Anzai A, Ohzawa I, Freeman RD (1999). Neural mechanisms for encoding binocular disparity: receptive field position versus phase.. J Neurophysiol.

[24] Prince SJ, Cumming BG, Parker AJ (2002). Range and mechanism of encoding of horizontal disparity in macaque V1.. J Neurophysiol.

[25] Bridge H, Cumming BG (2001). Responses of macaque V1 neurons to binocular orientation differences.. J Neurosci.

[26] Read JCA, Cumming BG (2003). Testing quantitative models of binocular disparity selectivity in primary visual cortex.. J Neurophysiol.

[27] Read JCA, Cumming BG (2007). Sensors for impossible stimuli may solve the stereo correspondence problem.. Nat Neurosci.

[28] Qian N (1994). Computing stereo disparity and motion with known binocular cell properties.. Neural Computation.

[29] Qian N (1997). Binocular disparity and the perception of depth.. Neuron.

[30] Adelson EH, Bergen JR (1985). Spatiotemporal energy models for the perception of motion.. J Opt Soc Am [A].

[31] Fleet D, Wagner H, Heeger D (1996). Neural encoding of binocular disparity: energy models, position shifts and phase shifts.. Vision Res.

[32] Dean AF (1981). The variability of discharge of simple cells in the cat striate cortex.. Exp Brain Res.

[33] Bair W, Koch C, Newsome W, Britten K (1994). Power spectrum analysis of bursting cells in area MT in the behaving monkey.. J Neurosci.

[34] Prince SJ, Pointon AD, Cumming BG, Parker AJ (2002). Quantitative analysis of the responses of V1 neurons to horizontal disparity in dynamic random-dot stereograms.. J Neurophysiol.

[35] Norcia AM, Tyler CW (1984). Temporal frequency limits for stereoscopic apparent motion processes.. Vision Res.

[36] Cogan AI, Lomakin AJ, Rossi AF (1993). Depth in anticorrelated stereograms: effects of spatial density and interocular delay.. Vision Res.

[37] Read JCA, Eagle RA (2000). Reversed stereo depth and motion direction with anti-correlated stimuli.. Vision Res.

[38] Tanabe S, Umeda K, Fujita I (2004). Rejection of false matches for binocular correspondence in macaque visual cortical area V4.. J Neurosci.

[39] Janssen P, Vogels R, Liu Y, Orban GA (2003). At least at the level of inferior temporal cortex, the stereo correspondence problem is solved.. Neuron.

[40] Cumming BG, Shapiro SE, Parker A (1998). Disparity detection in anticorrelated stereograms.. Perception.

[41] Stevenson SB, Schor CM (1997). Human stereo matching is not restricted to epipolar lines.. Vision Res.

[42] Prazdny K (1985). Vertical disparity tolerance in random-dot stereograms.. Bulletin of the Psychonomic Society.

[43] Cumming BG, Parker AJ (1997). Responses of primary visual cortical neurons to binocular disparity without depth perception.. Nature.

[44] Ohzawa I, DeAngelis GC, Freeman RD (1997). Encoding of binocular disparity by complex cells in the cat's visual cortex.. J Neurophysiol.

[45] Carandini M, Heeger D, Movshon T, U PS, J EG, P A (1999). Linearity and gain control in V1 simple cells.. Cerebral cortex: models of cortical function.

[46] Carandini M, Heeger DJ, Movshon JA (1997). Linearity and normalization in simple cells of the macaque primary visual cortex.. J Neurosci.

[47] Heeger DJ (1992). Normalization of cell responses in cat striate cortex.. Vis Neurosci.

[48] Simoncelli EP, Heeger DJ (1998). A model of neuronal responses in visual area MT.. Vision Res.

[49] Tolhurst DJ, Heeger DJ (1997). Comparison of contrast-normalization and threshold models of the responses of simple cells in cat striate cortex.. Vis Neurosci.

[50] Ayaz A, Chance FS (2009). Gain modulation of neuronal responses by subtractive and divisive mechanisms of inhibition.. J Neurophysiol.

[51] Matthews N, Meng X, Xu P, Qian N (2003). A physiological theory of depth perception from vertical disparity.. Vision Res.

[52] Garding J, Porrill J, Mayhew JE, Frisby JP (1995). Stereopsis, vertical disparity and relief transformations.. Vision Res.

[53] Backus BT, Banks MS, van Ee R, Crowell JA (1999). Horizontal and vertical disparity, eye position, and stereoscopic slant perception.. Vision Res.

[54] Gillam B, Lawergren B (1983). The induced effect, vertical disparity, and stereoscopic theory.. Percept Psychophys.

[55] Mayhew JE, Longuet-Higgins HC (1982). A computational model of binocular depth perception.. Nature.

[56] Leventhal AG (1983). Relationship between preferred orientation and receptive field position of neurons in cat striate cortex.. J Comp Neurol.

[57] Vidyasagar TR, Henry GH (1990). Relationship between preferred orientation and ordinal position in neurones of cat striate cortex.. Vis Neurosci.

[58] Bauer R, Dow BM (1989). Complementary global maps for orientation coding in upper and lower layers of the monkey's foveal striate cortex.. Exp Brain Res.

[59] Sasaki K, Tabuchi Y, Ohzawa I (2009). Complex cells in the early visual cortex have multiple disparity detectors in the 3D binocular RFs..

[60] Anzai A, Ohzawa I, Freeman RD (1997). Neural mechanisms underlying binocular fusion and stereopsis: position vs. phase.. Proc Natl Acad Sci U S A.

[61] Rogers BJ, Bradshaw MF (1993). Vertical disparities, differential perspective and binocular stereopsis.. Nature.

